# The Relationship between FFMQ Mindfulness and Harmony in Life among Patients with Celiac Disease

**DOI:** 10.11621/pir.2022.0103

**Published:** 2022-03-15

**Authors:** Emrullah Ecer

**Affiliations:** a Ural Federal University, Ekaterinburg, Russia

**Keywords:** FFMQ, mindfulness, celiac disease, harmony in life, duration of diagnosis

## Abstract

**Background:**

Patients with Celiac Disease (CD) experience psychological disorders and emotion-regulation disruptions. Although following a gluten-free diet alleviates their symptoms, these patients report social relationship problems.

**Objective:**

The first aim of this study was to analyze the level of FFMQ mindfulness (describing emotions, acting with awareness, observing, non-judging of inner experience, and non-reactivity to inner experience) and harmony in life (HiL) in patients with CD. The second goal was to examine the relationship between the FFMQ and HiL scales in patients with CD. The third was to detect the effects of the duration of the illness, education level, and employment status on FFMQ-measured mindfulness and HiL.

**Design:**

The study involved 111 Turkish patients with CD (N Females = 75, 67.6%) living in Turkey. The patients filled out the FFMQ and HiL questionnaires via a google form survey. The duration of their diagnosis, age, employment status, and education level were nominal variables. A Pearsons’ correlation test, independent t-test, multiple linear regression, and one-way ANO VA were implemented.

**Results:**

The results showed that patients with CD had a low level of HiL. The total FFMQ score was positively related to the HiL scale. Education and duration of diagnosis had a significant impact on the FFMQ and HiL scores. Age affected the level of describing emotions, and employment status had a strong effect on acting with awareness. However, gender affected neither the FFMQ nor HiL levels.

**Conclusion:**

The results showed that patients with CD expressed a low level of HiL. Non-reactivity to inner experience, observing, and acting with awareness were positive predictors of the HiL scores. Moreover, since the HiL and FFMQ scales showed high internal consistency, the FFMQ and HiL questionnaires can be used in further studies of patients with CD.

## Introduction

Celiac disease (CD) is an inflammatory, chronic immune-mediated disease characterized by persistent intolerance to gliadin in the small intestines (Rubio-Tapia, Hill, Kelly, Calderwood, Murray, & Murray, 2013). The prevalence of CD is 0.6 to 1.0% of the population worldwide (Singh et al., 2018). The incidence of CD has been increasing in many developing countries because of the westernization of the diet, changes in wheat production and preparation, and increased awareness of the disease (Fasano & Catassi, 2012).

Patients with CD have sensitivity to foods that contain gluten. A gluten-free diet alleviates the symptoms of the disease. However, it is difficult to detect this disease. Because some celiac disease symptoms resemble those of other diseases such as irritable bowel syndrome (IBS) and lactose tolerance (Makharia, Catassi, & Makharia, 2015), patients with CD face stressful conditions (Castilhos et al., 2015). In addition, gluten-free restaurants are limited. Therefore, patients with CD report problems around participating in social events. They often refuse to meet with their friends. During this time, their emotion-regulation is disrupted. Patients with CD feel frustrated and embarrassed due to their diet restrictions. They feel lonely and socially isolated (Ciacci et al., 2013). Their levels of depression and anxiety are related to emotion-regulation strategies. Cognitive reappraisal has been shown to be negatively related to depression in patients with CD (Kerswell & Strodl, 2015).

In addition to emotion-regulation difficulties, physical problems have been found in patients with CD. One of the most common complaints is feeling fatigued (Siniscalchi et al., 2005). Such symptoms may result in depressive tendencies and less adherence to a gluten-free diet (Campagna et al., 2017). A study conducted in Turkey found that patients with CD had a low level of quality of life, with participants reporting dysfunction in their social, emotional, and physical health (Sevinç, Çetin, & Coşkun, 2017). However, implementing a gluten-free diet decreased anxiety and depression symptoms, and raised the level of mental well-being (Wagner et al., 2015). Knowing about gluten-free products and the disease has a strong impact on mental well-being (Halmos et al., 2018). One study demonstrated that anxiety, depression, and fatigue were prevalent in patients whose CD was untreated, and thus induced a lower quality of life satisfaction (Zingone et al., 2015).

These mental health disruptions affect patients’ behavior and their family relationships (Martínez-Bermejo & Polanco, 2002). Symptoms of the disease affect the social activities and emotional states of the patients (Passanati et al., 2013). Patients with CD experience dissatisfaction with their body shape and weight (Arigo, Anskis, & Smyth, 2012). A narrative review analysis has indicated that eating disorders were positively associated with CD (Slim, Rico-Villademoros, & Calandre, 2018).

The effects of the disease are more serious in children. The research on children and adolescent samples between 7-18 years old has demonstrated that children with CD have higher scores for harm avoidance and somatic complaints. They tend to have more social, cognitive, and attention problems (Mazzone et al., 2011), and social phobias (Addolorato et al., 2008). Moreover, adults with CD tend to use a higher level of antidepressants than a control group (Zylberberg, Ludvigsson, Green, & Lebwohl, 2018). Their sense of social and emotional well-being is distorted (Al-Qefari, Al-Twijri, Al-Adhadh, Al-Rashed, & Al-Jarallah, 2018). It can be said having CD is a significant risk factor for psychological disorders.

One of the significant components related to the level of mental well-being in patients with CD is gender. Female patients perceive the burden of the disease more heavily than male patients do. They have lower levels of mental well-being than male patients with CD (Rodríguez-Almagro, Rodríguez-Almagro, Solano-Ruiz, Siles-González, & Hernandez-Martinez, 2019). In addition, women patients with CD report a higher ratio of gastrointestinal symptoms, even though they followed a gluten-free diet (Roos, 2011). Therefore, it can be said that women patients with CD are less resilient in the face of the impact of the CD than men with CD.

The second factor that affects the burden of the disease is the level of education. If patients complete higher education, they tend to be aware of the disease. Patients with higher education levels are less vulnerable to psychological problems (Leino-Kilpi et al., 2005). Furthermore, the duration of the disease plays an important role in determining the patients’ mental well-being. The first year of diagnosis has been shown to be a significant predictor of higher suicide risk in patients with CD (Lud-vigsson, Sellgren, Runeson, Långström, & Lichtenstein, 2011). On the other hand, the mental well-being of patients with CD who were treated for more than 10 years has not shown a significant difference in the level of anxiety, depressed mood, positive well-being, and self-control compared to a control group (Roos, Kärner, & Hallert, 2006).

In this case, integration with society has a significant role in determining mental well-being. Patients with CD need time to harmonize with their environments and adapt to novel life conditions. It has been reported that five years after diagnosis, patients with CD have fully adapted their skills in domestic and international travel. In the first two years after diagnosis, patients with CD still have problems explaining their disease (Clerx, Silvester, Leffler, DeGroote, & Fishman, 2019), and their subjective well-being is positively related to their levels of friendships and perception of support (Shani, Kraft, Müller, & Boehnke, 2020). Hence, harmonization with society may be disrupted during the first years of diagnosis in patients with CD.

It has been found that experiencing harmony in life (HiL) is negatively related to depression, anxiety, and stress, whereas it is positively correlated with subjective happiness, life satisfaction, psychological well-being, and social desirability (Kjell, Daukantaitė, Hefferon, & Sikström, 2016).

Furthermore, a positive interaction with society and family members might have an impact on the patients’ mindfulness score. One study found that the components of FFMQ mindfulness (describing, awareness, non-judging of inner experience, and non-reacting to inner experience, and observing) were negatively associated with depression, anxiety, and somatization (Cebolla et al., 2012). Another concluded that a high level of FFMQ mindfulness was negatively related to Alexithyhmia and depressed mood rumination (Lilja, Lundh, Josefsson, & Falkenström, 2013). A meta-analysis study reported that FFMQ-measured mindfulness had a negative relationship with generalized anxiety disorder and post-traumatic stress disorder. Acting with awareness and non-judging of inner experience were found to be negative predictors of social anxiety disorders (Carpenter, Conroy, Gomez, Curren, & Hofmann, 2019). Describing and non-judging of inner experiences were predicted to be related to the anxiety and depression symptoms with 7 percent of variance in patients with IBS, according to a study by Gaylord et al. (2011).

Overall, we can conclude that both FFMQ mindfulness and HiL can be significant variables in analyzing psychological well-being in patients with CD.

The main goal of this study was to determine the relationship between FFMQ and HiL scales in patients with CD. The second goal was to ascertain the effects of education level, age, duration of illness, and gender differences on the HiL and FFMQ levels.

There were several hypotheses to be tested in the study:

1. There will be a positive relationship between HiL and FFMQ in patients with CD.
2. Duration of diagnosis and education will have a significant impact on the HiL and FFMQ mindfulness in patients with CD.
3. Gender differences will have a significant effect on the HiL and FFMQ mindfulness in patients with CD.

## Methods

### Participants and Design

This study was carried out between March 23 and April 15 in 2020 in Turkey. Participants were contacted via CD Social Media Platforms. There were 111 participants (Females N = 75, 67.6%) who had been diagnosed with CD. Two individuals did not mark their gender. Age, duration of diagnosis, employment status, and education were nominal variables. The majority of the participants were over 30 years of age (N = 58, 52%), had been diagnosed with CD more than 5 years before (N = 53, 47.7%), and had a university degree (N = 41, 37%). Detailed information on the sample is shown in Table 1. Participants filled out the FFMQ and HiL scales via a google form. All participants had been diagnosed with CD for at least one year were included in the study.

**Table 1:** Demographic Variables

Variables	Frequency	Percentage
	N	(%)
*Gender*		
Female	75	67.6
Male	34	30.6
Missing	2	1.8
*Employment Status*		
Employed	53	47.7
Unemployed	32	28.8
Student	26	23.4
*Education*		
Primary	20	18.0
High School	27	24.3
University	41	36.9
Master	5	4.5
Students under 18 years	18	16.2
*Age*		
Over 30 years old	58	52.3
26–30 years old	15	13.5
18–25 years old	20	18.0
Under 18 years old	18	16.2
*Duration of Diagnosis*		
1–2 years	27	24.3
2–5 years	31	27.9
More than 5 years	53	47.7

### Procedure

#### Five Facets Mindfulness Questionnaire (FFMQ)

The FFMQ (Baer, Smith, Hopkins, Krietemeyer, & Toney, 2006) is a comprehensive scale of dispositional mindfulness and contains statements about our thoughts, experiences, and actions in daily life. In this study, the Turkish version of the FFMQ (Kınay, 2013) was used. In this version, the observing sub-scale has 9 items instead of 8, while the non-judging of inner experience sub-scale has 6 items instead of 7. The entire scale consists of 39 questions with five subscales, which participants were to evaluate numerically, with 1 = strongly disagree to 5 = “strongly agree.” The higher scores therefore reflect higher levels of FFMQ mindfulness. The five subscales are:

1. Observing/noticing/attending, which includes feelings, sensations, perceptions, thoughts, and sensory awareness of the internal and external world around us (e.g., “When I’m walking, I deliberately notice the sensations of my body moving”);
2. Describing, or the ability to label experiences and express them in words (e.g., “I’m good at finding words to describe my feelings”);
3. Acting with awareness and focusing on the information present at the moment (e.g., When I do things, my mind wanders off and I’m easily distracted);
4. Non-judging of inner experience, which includes accepting both one’s own and others’ thoughts, emotions, and the empathy statements (e.g., I criticize myself for having irrational or inappropriate emotions); and
5. Non-reactivity to inner experience, which refers to accepting one’s negative thoughts and emotions rather than internalizing them (e.g., I watch my feelings without getting lost in them)

In the current study, all scales showed adequate to high internal consistency (Omega coefficient values obtained, FFMQ = .80; observing = .81; acting with awareness = .92; describing = .85; non-judging of inner experience = .88; and non-reactivity to inner experience =.81).

#### Harmony in Life Scale (HiL)

The HiL questionnaire (Garcia, Al Nima, & Kjell, 2014) contains statements relevant to one`s perception, evaluation, and judgment relative to balance in one’s social relationships. The scale aims to evaluate one`s harmony and integration into both society and one’s self. In the current study, patients were scored on the Turkish version of the HiL scale (Satici & Tekin, 2017). The scale consists of five items (e.g., I accept different conditions in my life) ranging from 1 = Strongly disagree to 7 = Strongly agree. A higher score indicated a higher level of the HiL. In the current study, the scale had a high internal consistency (Omega coefficient = .87).

### Data Analysis

The data was computed by SPSS, version 26. The values of the skewness, kurtosis, and standardized z-scores of the kurtosis and skewness were measured to detect whether the study was normally distributed or not. It has been suggested that for medium-sized samples that range between (50<n<300), the standardized z-score of skewness and kurtosis between -3.29 and +3.29 (Kim, 2013) and between -2 and +2 (George & Mallery, 2010) indicates that the study is normally distributed. In this study, standardized z scores of skewness and kurtosis in all-subscales of FFMQ and HiL were found to be between -3.29 and +3.29 (see Table 2). Thus, the research was analyzed using parametrical tests.

**Table 2:** Descriptive Statistics

Scale	Mean	SD	Kurtosis (SE)	Skewness (SE)	Kurtosis (Z)	Skewness (Z)
HiL	23.1	7.7	–.08 (.46)	–.64 (.23)	–0.17	–2.78
FFMQ	124.5	19.2	.17 (.46)	–.02 (.23)	.37	–.09
Awareness	27.8	9.3	–.82 (.46)	–.38 (.23)	–1.78	–1.65
Describing	28.3	7.7	–.70 (.46)	–.24 (.23)	–1.52	–1.04
Observing	31.1	6.8	–.58 (.46)	–.50 (.23)	–1.26	–2.17
Nonjudge	19.8	7.8	–.05 (.46)	.61 (.23)	–1.08	2.65
Nonreact	17.5	5.5	–.38 (.46)	–.01 (.23)	–.082	–.004

*Note. SD = Standard Deviation; HiL = Harmony in Life; FFMQ = Total Dispositional Mindfulness; Nonjudge = Nonjudging of inner experience; Nonreact = Nonreactivity to inner experience; SE = Standard Error; Z = Standard Score*

This study had four independent variables – namely, gender, education, age, and duration of illness – with FFMQ and HiL as the two dependent variables. Pearson’s correlation test was used to detect the relationship between the components of the FFMQ (describing, observing, acting with awareness, non-judging of inner experiences, non-reaction to inner experiences) and HiL questionnaire.

An independent T-Test was computed to determine the effects of gender on the HiL and FFMQ scores. One-way ANO VA was measured to analyze the effects of education, employee status, age, and duration of illness on the HiL and FFMQ as well. Multiple Regression Analysis was used to examine the effects of FFMQ components on the HiL. In the second part of the Multiple Regression analysis, education and duration of illness were chosen as independent variables, while HiL and FFMQ were dependent variables. The reliability of the FFMQ and HiL scales was measured using McDonald`s Omega coefficient analysis (Flora, 2020).

The power of the sample size was analyzed using G*Power (Faul, Erdfelder, Buchner, & Lang, 2009). Since this power was computed after the study was completed, post hoc analysis was measured. The type of power for the Pearson’s correlation test was the bivariate correlation model of the post hoc two tails. The power of the linear multiple regression: fixed model, R2 deviation from zero was used to measure the sample size power of the multiple regression analysis.

## Results

The means of the HiL (M = 23.1, SD = 7.7) and dispositional mindfulness scores were obtained. For total FFMQ, M = 124.5, SD = 19.2; for acting with awareness, M = 27.8, SD = 9.3; for describing, M = 28.3, SD = 7.7; for observing, M = 31, SD = 6.8; for non-judging of inner experience, M = 19.8, SD = 7.8; and for non-reactivity to inner experience, M = 17.5, SD = 5.5.

To test the first hypothesis, the relationship between the FFMQ and HiL scores was analyzed. Pearson’s correlation analysis indicated that the HiL scale was positively correlated with the total FFMQ (r = .54, p = .0001), and the following subscales: observing (r = .41, p = .0001); describing emotions (r = .37, p = .0001); acting with awareness (r = .43, p = .0001); and non-reactivity to inner experience (r = .44, p = .0001). The HiL scale showed negative correlation with the non-judging of inner experience (r = –.22, p = .02). Therefore, it can be concluded that the first hypothesis was proven. Detailed analysis of the correlation test is in *Table 3*.

**Table 3:** Pearson’s correlation coefficients between scales

Variable	1	2	3	4	5	6	7
1. HiL	–						
2. FFMQ	.54***	–					
3. Awareness	.43***	.73***	–				
4. Describing	.37***	.71***	.34***	–			
5. Observing	.41***	.35***	–.01	.26**	–		
6. Nonjudge	–.22*	.21*	.17	–.12	–.47***	–	
7. Nonreact	.44***	.52***	.15	.38***	.29**	–.23*	–

Note. * = p<.05, ** = p<.01, *** = p <.001. HiL=Harmony in Life; FFMQ = Total Dispositional Mindfulness

Then, multiple regression analysis was computed to find the total variance of the FFMQ score on the HiL score. The results indicated that FFMQ mindfulness was explained with 41 percent total variance by observing, acting with awareness, and non-reactivity to inner experience: F (5, 105) = 16.12, p = .0001, R^2^ = .43. It was found that observing (B = .28, SE = .09, p = .001), acting with awareness (B = .39, SE = . 06, p = .0001), and non-reactivity to inner experience (B = .26, SE = .11, p = .002) were positive predictors of HiL. However, non-judging of inner experience and describing emotions were not predicted to be associated with the HiL scores.

**Table 4:** Multiple regression analysis measured the effects of FFMQ on HiL

Model	Unstandardized Coefficients		p	95.0% CI	
	Estimate	SE		LL	UL
Awareness	.32	.07	.0001	.19	.45
Nonreact	–.37	.11	.002	.14	.60
Observing	.31	.10	.001	.12	.51

*Note. DV = HIL; R2 = .43; Adj. R2 = .41; CI = Confidence Interval; LL = Lower Limit; UL = Upper Limit*

To test the second hypothesis, the effects of duration of diagnosis and education level were examined. Multiple regression results showed that HiL was explained with 6.5 percent variance by both education and duration of illness; F (2, 108) = 4.8, p = .01. R ^2^= .08. Duration of illness (B =.19, SE =.87, p = .04) and education level (B = .20, SE = .59, p =.03) were significantly related to HiL. FFMQ mindfulness was explained with 7 percent total variance by both education level (B = .21, SE = 1.5, p = .02) and duration of illness (B = .19, SE = .2.2, p = .04); F (2, 108) = 5.04, p = 0.008, R^2^= .09. Hence, it can be said that the second hypothesis was proven.

**Table 5:** Multiple regression analysis measured the effects of education and duration of diagnosis on FFMQ

Model	Unstandardized Coefficients		p	95.0% CI	
	Estimate	SE		LL	UL
Education	4.5	2.16	.04	.22	8.8
Duration of Diagnosis	3.4	1.48	.02	.51	6.4

*Note. DV = FFMQ; R2 = .09; Adj. R2 = .07; CI = Confidence Interval; LL = Lower Limit; UL = Upper Limit*

One–way ANO VA showed that education level had a significant impact on describing emotions; F (4, 106) = 3.03, p = .02). The mean level of describing by patients with CD who had masters’ degrees was *M* = 33, *SD* = 6.46; for those with university degrees, M = 29.5, SD = 6.9; for those with a high school degree, *M* = 30.1, *SD* = 6.7); for patients who were adolescent students, *M* = 26.4, *SD* = 9.7); and for patients with primary education, *M* = 24, *SD* = 7.48.

**Table 6:** Multiple regression analysis measured the effects of education and duration of diagnosis on HiL

Model	Unstandardized Coefficients		p	95.0% CI	
	Estimate	SE		LL	UL
Education	1.3	.87	.03	.09	3.5
Duration of Diagnosis	1.8	.59	.04	.14	2.5

*Note. DV = HiL; R2 = .08; Adj. R2 = .065; CI = Confidence Interval; LL = Lower Limit; UL = Upper Limit*

Duration of diagnosis had a significant effect on total FFMQ mindfulness; F (2,108) = 7.4, p =. 001). Patients with CD who had been diagnosed more than 5 years before (*M* =131, *SD* = 20) had a higher FFMQ score than patients diagnosed between 1-2 years before (*M* = 124, *SD* = 14) and patients who were diagnosed between 2-5 years before *(M* = 115, *SD* = 19).

Employment status affected the level of acting with awareness: F (2, 108) = 3.01, p = .05. Employed CD patients scored a higher level of acting with awareness (*M* = 30, *SD* = 8.36) than patients who were students (*M =* 26.5*, SD* = 10). Unemployed patients had the lowest scores on the acting with awareness scale (*M* = 25.1, *SD* = 9.69)

Age was a significant factor in the scores on describing emotions: F (3,107) = 2.9, p = .04. Patients between 18-25 years old had a higher level of describing score (*M* = 29.8, *SD* = 6.3) than patients who were older than 30 years old (*M* = 29.4, *SD* = 6.9), and adolescent patients with CD (*M* = 26.2, *SD* = 10). Patients between 26-30 years old had a lowest describing level (*M* = 23.4, *SD* = 7.6).

## Discussion

This study had two main objectives. The primary aim of the research was to examine the relationship between the CD patients’ levels of HiL and FFMQ mindfulness. The second goal was to identify the effects of duration of illness, age, gender, education, and employment status on their FFMQ and HiL levels. In addition, the study posed several hypotheses.

A previous study had reported that patients with CD were likely to complain of depressive symptoms (Smith & Gerdes, 2012). The current research suggested that patients with CD who had low HiL scores had similar scores to participants with high depression, anxiety, and stress symptoms (Satici & Tekin, 2017). The study found that the total FFMQ score (*M* = 114) for the adult depressed patients (Bohlmeijer, Klooster, Fledderus, Veehof, & Baer, 2011), patients (*M* = 112) with a social anxiety disorder (Makadi, & Koszycki, 2019), and patients with recurrent major depressive disorders before cognitive-behavioral therapy (*M* = 120) had a lower total FFMQ score than patients with CD (*M* = 124.5) (Gu et al., 2016)

FFMQ mindfulness plays an important role in the quality of relationships. A study by Goodall, Trejnowska, and Darling (2012) showed that anxious attachment was negatively correlated with describing, labeling with words, focusing in the present moment, non-judging of experience, and non-reactivity to inner experiences. This study found that HiL was positively related to total FFMQ, including acting with awareness, describing with words, observation, and non-reactivity to inner experience, whereas it was negatively associated with the non-judging of experience in CD patients. Moreover, 41% of the total variance of HiL was predicted by observation, acting with awareness, and non-reactivity to inner experience. It can be interpreted that paying attention to feelings, sensations, focusing on the present time, and accepting both one’s own and other people’s opinions have a high impact on integrating patients with CD into society.

It has been found that self-compassion has a significant impact on the quality of life and gluten-free diet adherence in patients with CD (Dowd & Jung 2017). On the other hand, patients with CD may have difficulty adapting to their gluten-free diet (Silvester, Weiten, Graff, Walker, & Duerksen, 2016). Previous research has suggested that the duration of diagnosis might affect the personality as well (Rosa, Troncone, Vacca, & Ciacci, 2004). The present study found that the duration of diagnosis had a high impact on FFMQ and HiL scores. When patients had been diagnosed with CD more than 5 years before, they reported a higher level of FFMQ and HiL scores. It can be highlighted that 5 years after diagnosis, patients with CD revive their social interactions and increase their mindfulness skills.

It has been reported that diagnosis of CD is not associated with the education level (Olen, Bigahen, Rasmussen, & Ludvigsson, 2016). However, education level, along with age, has been shown to have a high impact on psychological well-being (Belo, Navarro-Pardo, Pocinho, Carrana, & Margarido, 2020). The present study found that education level had a high impact on describing emotions. People with a higher level of education reported a higher level of expressing emotions. Furthermore, education, along with duration of illness, was positively associated with HiL and FFMQ mindfulness. Therefore, it can be said that a higher level of education for patients with CD might provide a protective effect for integration into society and mindfulness skills.

Age has been shown to be a significant factor in FFMQ mindfulness and psychological well-being (Hohaus & Spark, 2013). A previous study on patients with CD found that age did not have a significant effect on mood disorders and psychological well-being (Canova et al., 2021). However, the present study suggested that the patients’ age groups affected the level of their description of their emotions. Patients between the ages of 26-30 had particular difficulty expressing their emotions.

The mindfulness level has been shown to vary due to economic factors (Jensen, Krogh, Westphael, & Hjordt, 2019). When people had economic problems, they were less likely to access to the health care system that might have a significant impact on the diagnosis of CD (Roy et al., 2016). The present study showed that unemployed patients had a lower score on the acting with awareness scale than employed patients.

Previous research has reported that women patients with CD had a higher level of anxiety symptoms than men (Rostami-Nejad et al., 2020). However, in the current study, gender differences did not significantly affect either the mindfulness or HiL scores. Thus, it should be highlighted that the third hypothesis was proven false.

## Conclusion

This study found that HiL was explained with 41 percent of total variance by the FFMQ characteristics of acting with awareness, non-reactivity to inner experiences, and observing. However, observing and non-judging of inner experiences were not predicted to be related to HiL. Moreover, Pearson’s correlation test also showed that HiL was positively correlated with the dispositional mindfulness characteristics of acting with awareness, describing, observing, and non-reactivity to inner experiences, whereas non-judging of inner experiences was negatively associated with the HiL.

The study also suggested that education level and duration of illness were associated positively with HiL and FFMQ. One-way AN O VA data showed that duration of illness had a significant impact on FFMQ mindfulness; patients diagnosed with CD more than 5 years before scored a higher level of FFMQ. In addition, education level and age affected the scores on describing emotions. Patients with masters’ degrees and those between 18 and 25 years of age had higher scores on describing emotions.

The study found that gender differences did not have a significant impact on the HiL and FFMQ scores. While the research has suggested that people with CD have a low level of HiL, the current findings on the FFMQ’s association with psychological disorders remains questionable. Therefore, in future studies, it would be beneficial to evaluate the relationship between FFMQ, anxiety, depression, and social anxiety symptoms in patients with CD.

## Limitations

This study had several limitations. The first was that it was the cohort study survey that computed the relationship between variables rather than attempting to predict outcomes. Further studies may focus on an experimental design that examines the effects of cognitive-behavioral therapy on FFMQ mindfulness. The second limitation was the sample size. G* Power analysis indicated that the correlation sample size between HiL and non-judging of inner experiences (.65), and the multiple regression analysis sample size that examined the relationship duration of diagnosis and education level on FFMQ and HiL (.69), had a lower effect size than 80%. However, multiple regression analysis between the FFMQ and HiL had an excellent power sample size (.98).

The third limitation was that, when the adjusted Bonferroni p alpha value was computed, the significant effects of one-way ANO VA data statistics disappeared. Therefore, the effects of duration of diagnosis, employment status, age, and education level should be determined in further studies. Moreover, the representation of males and females in the ample was unbalanced (*N* Female = 75, Male = 34). Further studies should have a larger sample with equal gender groups.

The fifth limitation was that the research did not have a variable that assessed whether the participants followed a strict gluten-free diet. Therefore, it would be interesting to evaluate whether the FFMQ and HiL levels have been linked to gluten-free diet adherence.

The sixth limitation was that FFMQ and HiL scales were self-report measures, which therefore poses a risk for potential bias. Yet, there has been no previous study based on the FFMQ and HiL scales in patients with CD. The FFMQ and HiL scales had high internal consistency scores in patients with CD and can be used in further studies.
